# Reports of Cases Occurring in the Medical Ward of the Buffalo Hospital of the Sisters of Charity, during the Session of the Medical College for the Winter of 1856—7

**Published:** 1857-04

**Authors:** A. W. Nichols

**Affiliations:** Assistant in the Department of Clinical Medicine


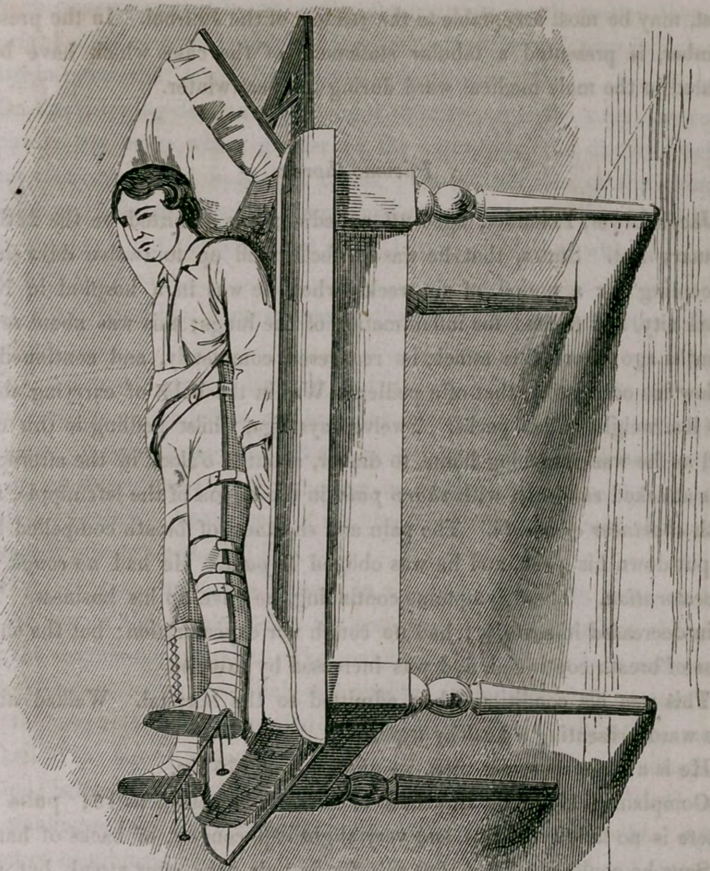

ART. III.—*Reports of Cases occurring in the Medical Ward of the Buffalo Hospital of the Sisters of Charity, during the Session of the Medical College for the Winter of* 1856—7. *Dr. A. Flint, Prof. of Clinical Medicine and Pathology, Attending Physician.* Reported by the Assistant in the Department of Clinical Medicine, Dr. A. W. NICHOLS.

*Prefatory.* These reports, which are to appear monthly in the Journal, will consist of the previous history, progress, and termination of the most interesting cases which have come under the notice of the medical class during the past session of the college: including remarks on the all important and practical points presented in each case. From the great variety of diseases which have occurred in the ward, such subjects will be selected, as we trust, may be most acceptable to the readers of the Journal. In the present number, is presented a tabular statement of the cases which have been treated in the male medical ward during the past winter.

*Pneumo-thorax.*

Jacob Liwer, Polander, aged 29, a pedler; was admitted on the 24th of January last. States, that he was perfectly well up to twelve days since, excepting for a period of six weeks, when he was in a hospital in New York city, and treated for inflammation of the lungs; this was about seven months ago; from this attack, he recovered completely, and continued to follow his occupation, that of a pedler. Was in the habit of carrying about 70 lbs. weight in his packs. Twelve days ago, whilst pedling in this city, and as he was returning home to dinner, about 2 o'clock in the afternoon, was attacked *suddenly*, with *sharp pain* in the region of the left nipple, and with *shortness of breath.* The pain and shortness of breath compelled him to put down his pack, and he was obliged *to pant.* He had *no cough nor expectoration.* These symptoms continuing, he gave up his business. The pain decreased in severity; had no cough nor expectoration; but the shortness of breath continued, and was increased by exercise.

This was his condition when admitted to the hospital. Walked about the ward presenting a healthy aspect.

He is a large muscular man, weighing about 175 lbs.

Complained alone of shortness of breath; respirations 16; pulse 92. There is no lividity of lips and very slight cap. congest. of backs of hands.

Says he could not lie on *left* side for first six days after attack, but now he can lie for a short time on that side.

Physical exploration of chest furnished the following signs:

Left side, in front, full and rounded at summit; less mobility at left summit. The *intercostal depressions* on left side, laterally and anteriorly, are *obliterated*, not visible on forced breathing, while they are distinctly so on the right side. The two sides measure, below the nipple, each 18½ inches. He is right handed. The spine is curved forward, not laterally. Measured from behind forward or transversely, right side is 7¾ inches; left side, 8¼ inches.

Percussion-resonance at left summit, clear and, relatively, to right vesiculo-tympanitic. *No respiratory sound* at left summit nor behind on left side. Well evolved vesicular murmur on right side, in front and behind.

No râles anywhere.

Very *clear resonance* in precordia; sonorousness much greater than on the right side; *no apex impulse of heart* anywhere; sounds very indistinctly heard in præcordia; heard distinctly in epigastrium.

On the following day, on examining with reference to the voice, the vocal resonance was every where greater on the right side, the disparity being about that often found in healthy chests. On the left side, at the summit, in front and behind, a distinct, almost perfect, *metallic echo* was heard both with the loud and the whispered voice, but more marked with the *latter* than with the former.

On the 27th of Jan., another examination of the chest afforded the following signs:

The intercostal spaces, laterally and anteriorly, on left side, are *now equally visible* on the left as on the right side; the left nipple is somewhat elevated; on measuration, the left side is 19 and the right 18½ inches.

The left side is every where *sonorous* on percussion, the *quality* of sound being *tympanitic.* At the base of the chest, behind and in front, the sonorousness is greater on the left side.

*Amphoric respiration* was present in a marked degree, in front, on left side, as low as the nipple, below the scapula behind; it was not observed over the scapula nor laterally. The sound was of a *silvery tone,* and every where the same as regards pitch and quality; its intensity was greatest below the scapula; it accompanied the *inspiration* and *expiration,* more marked in the latter; it appeared *distant,* not near the ear, in any situation. Behind, below left scapula, every where, *metallic tinkling* was heard; several tinkling sounds follow *expiration,* not uniformly, but frequently. After an act of coughing, several tinkling sounds occur. They are not observed after speaking.

The amphoric sound accompanies the acts of speaking and whispering, but is prolonged after these acts: it is not, therefore, *strictly* an echo; it is more marked during the act of whispering than speaking: the tone is uniformly silvery.

At the *summit* of the chest, in front, a very distant, scarcely appreciable, low *respiratory sound* is heard. *Just below the left scapula,* a feeble, but distinct, low, *vesicular murmur* was heard during this examination; on taking a *deep inspiration,* or *coughing slightly,* the *amphoric sound* was developed.

The treatment of the patient was palliative merely; he was ordered to avoid exercise, to remain in the ward, and was placed on the use of gum ammoniac mixture, ℥ss. three times.

On the 30th of Jan., the same signs were exhibited: the respiratory murmur, vesicular in quality, however, was heard more distinctly on left side, behind.

February 1st. The patient still presents the same physical signs. With reference to the heart, the *sounds were not heard in the precordia;* the *maximum* of the sounds was on the *right side*, between the nipple and the sternum; they were heard also in the epigastrium, louder to the right than to the left of the ensiform cartilage. The *pulsation of heart* was seen and felt *in epigastrium*, but not in the precordia.

The patient has no cough nor expectoration, nor any pain in the chest; complains only of *want of breath on exercise;* walks daily out of doors, a considerable distance; presents an healthy aspect. He thinks that he experiences less want of breath, on exercise, than when he entered hospital.

Feb. 10th. This patient kept the bed this morning, complaining of pain, not severe, at the lower part of the left side; no febrile movement; no cough.

The sonorousness on the left side still extends to the base of the chest. Below the ribs, in front, the gastric, tympanitic quality of resonance is marked over the chest; the gastric pitch of sound does not extend over the ribs.

The left side appears enlarged and rounded. The intercostal depressions are less marked on deep inspiration; left nipple is elevated; mobility of left side is relatively less; left side appears to be constantly distended to the limits of a full inspiration. *No respiratory sound*, in front, *on the left side*, anywhere, except at the summit; the respiration, tracheal or bronchial, is distant and feeble. *Metallic respiration nowhere heard; nor any metallic tinkling;* at the base, behind, left side, *metallic whisper still heard.*

On the 16th of Feb., a marked improvement had taken place, and the patient could take some amount of exercise without experiencing any want of breath. A change, likewise, was found in examining the chest.

The left side was less full and rounded. The intercostal depressions *equally marked* on the two sides. More mobility on right side.

Percussion-resonance on the two sides presented but *little disparity;* left side resonant even to base. Heart sounds still heard to the right of sternum and not on left side, their maximum being *directly to the right of the median line* of the nipple and on transverse line of nipple.

Respiration on right side, every where, was intense and vesicular. On left side, in front, it was heard as low as the nipple: it was quite feeble, low, and distant: behind, only appreciable and faint below scapula.

Greater vocal resonance on right side, every where; *no metallic intonation* during the spoken or whispered words; *no metallic tinkling.*

On Feb. 22d, reported better. Examination showed a diminution in amount of air in pleural sac.

Feb. 28th. Patient daily improving and experiencing less the want of breath on moderate exercise.

Maximum of heart's impulse is over sternum just above the xiphoid cartilage.

On semi-circular measurement, right side is half an inch larger than the left.

March 9th. This patient leaves the hospital to-day, intending to resume his former occupation. His aspect is healthy; complains of no pain, no cough, no expectoration; appetite good; does not experience deficiency of breath on moderate exercise. The physical signs are as follows:

Tympanitic resonance on left side; no distinct respiratory sound, in front, on left side; exaggerated vesicular murmur on right side; behind, no expiratory sound over left scapula; at base of left side distinct, vesicular murmur less intense than on the right side, and higher in pitch.

*Maximum of heart's impulse is to left of the ensiform cartilage.* The sounds are heard between the *median line and vertical line of left nipple.*

The treatment has been entirely palliative, or rather expectorant. Of late, a simple liniment was applied to chest as a placebo.

*Remarks.* This case affords many points of interest. Dr. Flint supposes that in this instance an emphysematous bleb of the pleura pulmonalis was ruptured, and the air being thereby admitted into the pleural sac, gave rise to the sudden and severe pain, and the great deficiency of breath. This theory would also account for the presence of most of the physical signs. The sudden manner of the attack would lead to the belief that perforation of the pleura pulmonalis occurred at that time. A considerable amount of air was admitted into a serous structure, the pleura. Pain and deficiency ot breath, instantaneous and so great as to cause the patient to sit down his bundles and pant for breath, appeared. No cough, no expectoration, no febrile movement, followed. In fact, no inflammation was established. No inflammation having occurred, none of its products as fibrin or liquor sanguinis could have been present. There was then air alone in the pleura; this was shown by the greater sonorousness of the left side, by the tympanitic quality of the sound, everywhere, even to the base behind. The amount of air was considerable, for it compressed the lung and forced it upward and backward, and also removed the heart from the left to the right of the sternum. The former was shown by the absence of the respiratory sound, except at the summit of the chest, in front and behind. The latter by the impulse of the heart in the epigastrium, and by the maximum of the sounds being heard to the right of the sternum. The air soon was absorbed in part, and when the patient enters the hospital, in addition to the above points, he presents most beautiful examples of *amphoric respiration;* of *metallic sound* accompanying and following both loud and whispered words, not truly an *echo;* and lastly, of metallic tinkling. It would, perhaps, be difficult to account for this last sign. Here we have great sonorousness on the left side, everywhere; and behind, everywhere, below scapula, metallic tinkling distinct and unmistakeable. This combination of physical signs is very singular: of course, very rare; and for this, probably, the more important. A certain quantity of liquid has been supposed to exist, almost always, where this sign has been discovered; air must necessarily be present. This sign has been considered almost pathognomonic of pneumo-hydrothorax. In this case are presented all the phenomena of that disease, excepting any sign to verify the existence of liquid, as dullness or flatness on percussion; we have, on the contrary, an increase, every where, of the sonorousness on the affected side. On these points, more fully, see "Flint on the Respiratory Organs," pages 282 *et seg* and 587 *et seg.*

Whilst the patient remains in the hospital, great care being taken with him as to any imprudence in exercise, the air disappears slowly, is gradually absorbed; and *pari passu* with its absorption, we have the corresponding physical signs on examining the chest. There is less difference as regards size, mobility, and general appearance, on the two sides. The great sonorousness of left side diminishes; the quality of sound becomes less tympanitic; the amphoric respiration fades away; the beautiful, silvery-toned, incomplete echo grows less and less distinct, and, finally, is lost. The metallic tinkling is heard less frequently; and hard coughing, or sudden change of posture, must be made to produce it. The natural respiratory sound is heard, now and then, at the summit at first, then occasionally, on left side, behind, just below the scapula. The heart also gradually returns to the left side, with the absorption of the air; we find the impulse and maximum of sounds to denote its gradual progress to the left.

The patient himself experiences the same favorable change. He can walk farther without discomfort; he suffers much less from shortness of breath. At last he is able to take considerable exercise, and leaves the hospital to resume his former avocation.

In reference to the admission of air into serous structures, and the danger of exciting inflammation thereby, we purpose to write more at length in the future.

